# Silk Fibroin-Based Biomaterials for Biomedical Applications: A Review

**DOI:** 10.3390/polym11121933

**Published:** 2019-11-24

**Authors:** Thang Phan Nguyen, Quang Vinh Nguyen, Van-Huy Nguyen, Thu-Ha Le, Vu Quynh Nga Huynh, Dai-Viet N. Vo, Quang Thang Trinh, Soo Young Kim, Quyet Van Le

**Affiliations:** 1 Laboratory of Advanced Materials Chemistry, Advanced Institute of Materials Science, Ton Duc Thang University, Ho Chi Minh City 700000, Vietnam; nguyenphanthang@tdtu.edu.vn; 2 Faculty of Applied Sciences, Ton Duc Thang University, Ho Chi Minh City 700000, Vietnam; 3 Institute of Research and Development, Duy Tan University, Da Nang 550000, Vietnam; nqv031187@gmail.com; 4 Key Laboratory of Advanced Materials for Energy and Environmental Applications, Lac Hong University, Bien Hoa 810000, Vietnam; vhnguyen@lhu.edu.vn; 5 Faculty of Materials Technology, Ho Chi Minh City University of Technology (HCMUT), Vietnam National University–Ho Chi Minh City (VNU–HCM), 268 Ly Thuong Kiet, District 10, Ho Chi Minh City 700000, Vietnam; lthuha2410@gmail.com; 6 The Faculty of Pharmacy, Duy Tan University, 03 Quang Trung, Danang 550000, Vietnam; quynhngaenglish@gmail.com; 7 Center of Excellence for Green Energy and Environmental Nanomaterials (CE@GrEEN), Nguyen Tat Thanh University, 300A Nguyen Tat Thanh, District 4, Ho Chi Minh City 755414, Vietnam; daivietvnn@yahoo.com; 8 Cambridge Centre for Advanced Research and Education in Singapore (CARES), Campus for Research Excellence and Technological Enterprise (CREATE), 1 Create Way, Singapore 138602, Singapore; qttrinh@ntu.edu.sg; 9 Department of Materials Science and Engineering, Korea University, 145 Anam-ro, Seongbuk-gu, Seoul 02841, Korea

**Keywords:** silk fibroin, drug delivery, biologics delivery, wound healing, bone regeneration

## Abstract

Since it was first discovered, thousands of years ago, silkworm silk has been known to be an abundant biopolymer with a vast range of attractive properties. The utilization of silk fibroin (SF), the main protein of silkworm silk, has not been limited to the textile industry but has been further extended to various high-tech application areas, including biomaterials for drug delivery systems and tissue engineering. The outstanding mechanical properties of SF, including its facile processability, superior biocompatibility, controllable biodegradation, and versatile functionalization have allowed its use for innovative applications. In this review, we describe the structure, composition, general properties, and structure-properties relationship of SF. In addition, the methods used for the fabrication and modification of various materials are briefly addressed. Lastly, recent applications of SF-based materials for small molecule drug delivery, biological drug delivery, gene therapy, wound healing, and bone regeneration are reviewed and our perspectives on future development of these favorable materials are also shared.

## 1. Introduction

In addition to cellulose, chitosan, and collagen, silk is one of the most abundant naturally derived polymers. Moreover, silk has been well-known as a luxury raw material in the textile industry for thousands of years since the first discovery in Chinese and Indus civilizations nearly 2500 BC [1,2]. Owing to the development and spreading of sericulture, where silkworms are reared and fed with mulberry leaves to collect silk fibers from their cocoons, silk fabrics have been produced on a large scale across South Asian countries ever since. This luxury textile material led to the establishment of the Silk Road, the great trading route that connected a part of Asia with regions in Europe, India, and Africa [3]. Owing to its unique lustrous appearance, tactile properties, durability, dyeability, excellent mechanical strength, flexibility, breathability, and comfort it provides in warm or cold weather, silk is historically acclaimed as the queen of textiles. More than 120,000 metric tons of silks are produced globally every year, and the main manufacturers are located in China, India, and Japan [4]. In addition to the abovementioned characteristics, biocompatibility also allowed the use of silk as a surgical suture since 150 AD [5]. However, we are only beginning to discover the real potential of this sophisticated material in more advanced application fields, such as optics, electronics, and biomedicine. The advanced technologies and modern tools used in chemistry, material engineering, chemical engineering, genetic engineering, and conceptual modeling have contributed significantly to analyzing the structure and extraordinary properties of silk. Owing to its hierarchical structure and versatility, many researchers have focused on silk as a model, bio-based candidate for designing novel materials with customized properties and remarkable performance for a diverse range of specific applications, such as wearable electronic devices, water ultrafiltration systems, biosensors, drug delivery systems (DDSs), and tissue engineering applications [6,7,8,9,10]. To achieve the full potential of silks for such advanced applications, fundamental material knowledge and familiarity with physics, chemistry, and biology concepts are highly required. Furthermore, nanoscience and nanotechnologies would play a significant role in the applications of this ancient material to modern technological processes.

**Figure 1:**
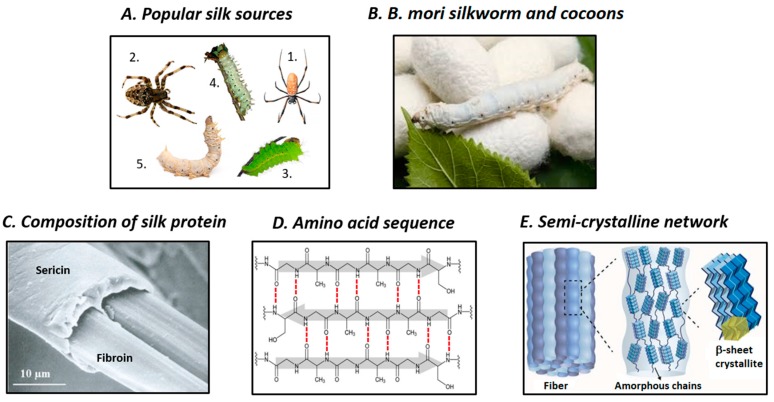
Overview of the origin and structures of silk fibroin. (**A**) Popular silk sources include *Nephila clavipes* (1.) and *Araneus diadematus* (2.) spiders, *Antheraea pernyi* (3.) and *Samia cynthia ricini* (4.) wild silkworms, and *Bombyx Mori* (5.) domestic silkworms. (**B**) Among them, *B. mori* silkworm is the most dominant source for silk fibers production. (**C**) Main proteins of silkworm silk fibers are fibroin and sericin (reproduced with permission [15]). (**D**) Hydrogen bonds between primary amino acid sequence of fibroin contribute to the generation of β-sheet crystallites (reproduced with permission [16]). (**E**) Fibroin is assembled from nanofibril units which crystal network consists of β-sheet crystallites dispersed within an amorphous matrix (reproduced with permission [17]).

Silks are defined as a unique class of biopolymers that are products of the secretion ‘spinning’ process of a number of arthropod lineages who use them to build their nests (honey bees and wasps), cocoons (silkworms), and webs (spiders) [11]. In addition to over 30,000 known species of spiders, most of the 113,000 known species of *Lepidoptera* can produce silks [12]. Furthermore, crickets, bees, wasps, fleas, lacewings, caddisfly larvae, aquatic midge larvae, glowworms, and fungus gnats are also known to produce silks [4]. However, the most productive sources of silks are the *Nephila clavipes* and *Araneus diadematus* spiders, *Bombyx mori* (*B. mori*) domestic silkworms, and *Antheraea pernyi* and *Samia cynthia ricini* wild silkworms [4,13] (Figure 1A). The mechanical strength of spider silks is superior to that of silkworm silk. However, the availability of spider silks is limited, and consequently, domestic *B. mori* filaments are the most commonly used in the commercial silk industry [14]. *Bombyx mori* (Figure 1B) silks are typically produced via a cycle that consists of different stages [4]. First, silkworm eggs are laid and incubated in a controlled and disinfected environment for 10 days prior to larvae hatching. Subsequently, the larvae are nourished with good quality, chopped mulberry leaves for six weeks. Then, the larvae spin fibers to form cocoons that protect them against microbes, moisture, and predators during metamorphosis. Mid-metamorphosis, *B. mori* silkworms are killed before they transform into pupae and the cocoon fibers are unraveled into commercial silk fibers. Hereafter, *B. mori* silkworm silks will simply be termed as “silk”. In this review, we describe the structure, composition, general properties, and structure-properties relationship of silk fibroin (SF), the main protein of silk. In addition, the methods for fabricating various silk-based materials are briefly described, and SF-based materials for drug delivery, bone tissue engineering, and wound healing are introduced. Lastly, our perspectives on the future development of these materials are also presented.

## 2. Structure and Properties of SF

At macroscopic level, natural silkworm silk thread comprises two structural proteins: fibroin (72–81 wt%) and sericin (19–28 wt%), and also small amounts of fat/wax (0.8–1%) and color/ash (1–1.4%). Fibroin, the main component of silk, acts as the inner core and provides mechanical strength, while sericin is the outer glue-like coating. Each silk fiber contains two SF filaments coated with sericin (Figure 1C) [18]. It has been proposed that SF filaments are assembled from nanofibrils that are 3–5 nm in diameter, which are the building blocks of silk. These nanofibrils interlock, interact strongly with each other, and assemble into larger fibril units that are 20–200 nm in diameter, which are known as microfibrils [19]. Microfibrils and nanofibrils arrange parallel to SF filaments. The strong friction between the twisted bundles of nanofibrils is the main reason for the strong interactions, and causes the excellent mechanical strength of silk fibers.

Silk fibroin, the main structural protein of silk, contains polypeptide chains with molecular weight in the range of 200–350 kDa. The primary structure of SF comprises repetitive blocks of hydrophobic heavy chains (H-fibroin, *M*_w_ = 391.6 kDa) and hydrophilic light chains (L-chain, *M*_w_ = 27.7 kDa) with terminal C and N groups. The linkers between these chains are disulfide bonds. In addition, glycoprotein P25 (*M*_w_ = 25.2 kDa), which is non-covalently linked to the above-mentioned chains is also present in the structure of SF and provides integrity to the entire structure. The molar ratio of H-fibroin, L-fibroin, and P25 in silk is 6:6:1 [20]. Hydrophilic l-fibroin comprises a small number of amino acid sequences: 14% alanine (Ala), 10% serine (Ser), 9% glycine (Gly), and acetylated N-terminal Ser residues, while hydrophobic H-fibroin consists of 45.9% Gly, 30.3% Ala, 12.1% Ser, 5.3% tyrosine (Tyr), and 1.8% valine (Val) [21]. The amino acid sequence of H-fibroin can be described as (–Gly–Ser–Gly–Ala–Gly–Ala–)_n_. Hydrophobic H-fibroin chains consist of repetitive hydrophobic domains interspersed between non-repetitive hydrophilic domains [20,22]. The repetitive hydrophobic amino acid domains of H-fibroin fold and bond together via hydrogen bonds, Van der Waals forces, and hydrophobic interactions, to form anti-parallel β-sheet crystalline structures (Figure 1D). These crystalline domains are highly organized at microscopic level and act as crosslinking points in the less ordered, poorly oriented amorphous matrix, which comprises random coils, β-turns, and/or α-helix structures and is formed of non-repetitive domains; the end result is a molecular fishnet structure (Figure 1E) [17,23]. The fishnet, semi-crystalline structure could be considered to be the fundamental architecture of silk nanofibrils. The strong β-sheet interactions, high degree of ordering, and high density of β-crystallites are believed to absorb impact pressure and distribute it throughout the entire fibroin network, and thus, confer excellent mechanical strength to the silk network. The secondary structures of silk could be classified into three crystalline forms: silk I, II, and III [17,24,25]. Of these, silk I is the liquid, metastable form of SF that is stored in the glands of silkworms and has been described as a partially ordered structure that could contain α-helix and even random coil structures. Conversely, silk II is the solid SF that forms after spinning, which features β-sheet crystalline structure. Lastly, silk III is a solid form of SF that features mostly trifold helical chain conformation and is found at the air/water interface [25]. During the spinning of silkworm silk, its conformation changes from dissolved, less-ordered silk I to solidified, highly ordered silk II, which results in the formation of silk fibers [4]. Controlling the internal secondary structure of silk is a powerful method for sophisticatedly adjusting its external properties, such as mechanical strength, solubility, and biodegradability.

**Figure 2:**
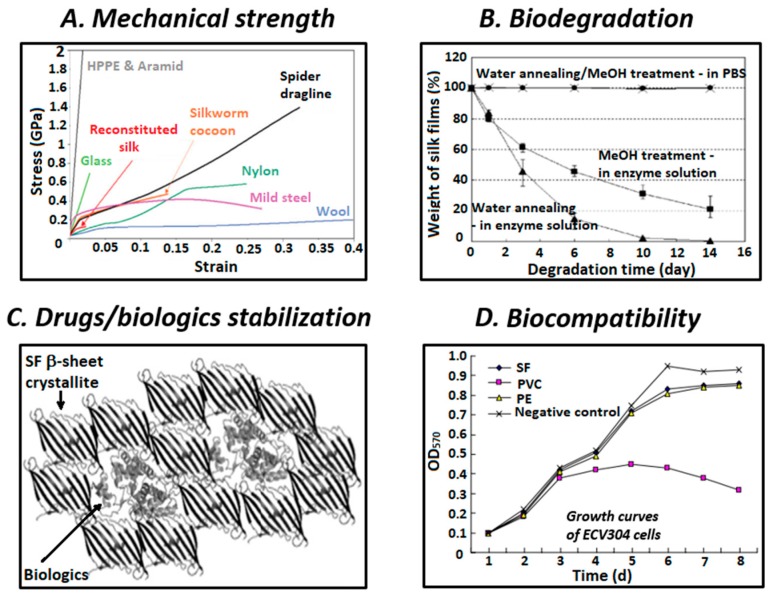
Key advantageous properties of silk fibroin for biomedical applications include: (**A**) robust mechanical strength with high tensile strength, modulus, stiffness, and extensibility (reproduced with permission [11]); (**B**) enzymatic biodegradation with controllable rate (reproduced with permission [26]); (**C**) payloads stabilization capability due to hydrophobic interactions with β-sheet crystallite domains (reproduced with permission [27]); and (**D**) biocompatibility proved by normal growth of ECV304 cells with no adverse influence after culturing with silk fibroin (SF) films (reproduced with permission [28]).

Owing to its mesoscopic hierarchical protein structure, silk possesses many desirable properties, and therefore is a promising material for future applications in a diverse range of areas, especially for biomedical applications (Figure 2). The hierarchical structure, spinning conditions, and amino acid sequences of silk are determining factors for the biological and physical properties of different types of SF [29]. To understand the structure-process-properties relationship of silk fibers, the combination of modeling tools and experimental characterization methods is highly required [30]. In general, owing to the embedded nanofibrillar structure, silks exhibit excellent mechanical properties and perfect balance between their strength, modulus, toughness, extensibility, light weight, and flexibility [31]. Despite the light weight of silk, its tensile strength is superior to those of other biopolymers, such as collagen and poly(L-lactic acid) and is comparable to those of nylon and mild steel (Figure 2A) [15,18]. Furthermore, the toughness of silk fibers is higher than that of Kevlar [32]. Hence, the strength-to-density ratio of silk is very high, which renders silk suitable for applications that require the combination of high strength and low density. This could be attributed to the high volume of highly organized β-sheet crystallites being responsible for the high tensile strength and modulus of silk, while the supporting amorphous bridges provide its lateral strength and elasticity [17,23,33]. The environmental stability of silk is also excellent. Owing to its high crystallinity and intrinsic hydrophobicity caused by a large amount of intra- and intermolecular hydrogen bonds, SF is insoluble in most organic solvents and water [34]. Nonetheless, aqueous solutions of SF can still be obtained via regeneration. Moreover, the thermal stability of SF is also a useful property, particularly for optoelectronic applications. Silk fibroin film has been reported to be thermally stable up to approximately 200 °C, where the side chain groups of amino acid residues break down and the peptide bonds decompose [35]. The excellent environmental stability of SF is attributed to the multiple hydrogen bonds between repetitive amino acid sequences, its hydrophobic nature, and high degree of crystallinity [36].

Biocompatibility is considered to be one of the most important requirements of materials used for biomedical applications. Many preclinical studies have reported that silk is a “clinically approved” biomaterial for human use. Nevertheless, biocompatibility is not universal and biocompatibility requirements depend on particular circumstances [37]. The clinical approval of silk-based biomaterials has been limited because some adverse immunological events associated with the use of silk protein could not be ignored [38]. Some researchers focused on the in depth examination of the immunogenicity and antigenicity of silk scaffolds, and reported no signs of infection and only minimal inflammation or very mild immune responses [39,40,41]. The outer sericin coating of silk could lead to delayed hypersensitivity reactions. Moreover, the role of sericin in silk-caused adverse effects has been recently debated because it was demonstrated that sericin could induce allergic reactions, immunogenicity, and the release of the tumor necrosis factor alpha inflammatory marker in a similar degree compared to fibroin [42,43]. The recent research into sericin for biomedical applications has yielded positive early results. It has been suggested that regardless of the presence of sericin, other leachable compounds could be the main cause for the adverse effects caused by silk [44,45]. However, the removal of sericin via a degumming process is still an essential step for SF processing. Degummed SF induced lower immunogenic response than other common biomaterials, such as synthetic poly(lactic-co-glycolic acid) (PLGA) and even collagen, which indicated its acceptable biocompatibility [39].

Considering the processes biomaterials participate in when implanted in the body, while biocompatibility is required to ensure the safety when applying and during administration period, biodegradation is also required for the complete clearance of the implanted device after service. The degradability of silk is driven by the proteolytic degradation mechanism and can be precisely controlled by changing its processing parameters and also its crystallinity (Figure 2B) [38]. Particularly, the degradation time of SF depends on various factors, such as external pressure, material content and format, secondary structure, treatment conditions, and characteristics of implanted site [29]. Biodegradability is the biological-caused disintegration and removal of implanted materials, while bioresorbability is the complete clearance of implanted materials via filtration or metabolization. The in vivo investigation of Wang et al. suggested that SF scaffolds are not only biodegradable within a few weeks but also bioresorbable after one year [41]. The degradation of SF occurs via enzymatic surface-erosion, unlike that of other synthetic polymers, such as PGLA, which undergo bulk-hydrolysis degradation [44]. Owing to its slow loss of mechanical strength, non-toxic by-products and controllable degradation rate, SF presents distinct benefits for biomedical applications, compared with other synthetic or natural polymers [46].

## 3. Processing of SF Biomaterials

**Figure 3:**
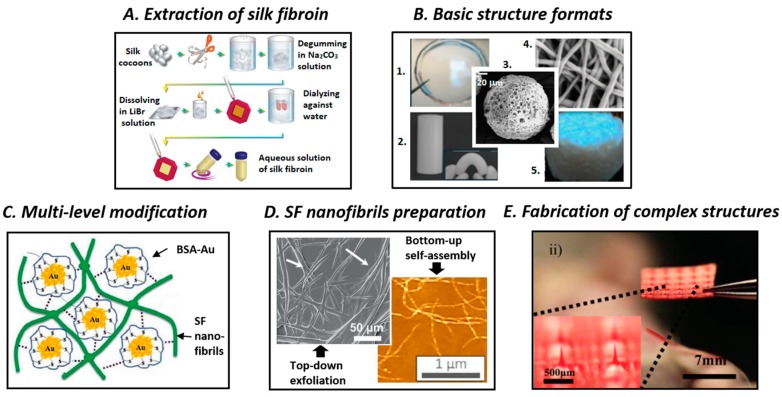
Overview of SF-based materials fabrication and modification. (**A**) Aqueous solution of silk fibroin can be obtained from silk cocoons through degumming, rehydration, and dialysis steps (reproduced with permission [47]). (**B**) Basic structures of SF-based materials include film (1.), hydrogel (2.), micro/nanoparticles (3.), fibers (4.), and scaffold (5.) (reproduced with permission [50,51]). (**C**) Functional SF-based materials can be designed via multi-level modification techniques, for example, self-assembly of SF nanofibrils network mediated by bovine serum albumin-gold (BSA-Au) nanocluster complex (reproduced with permission [52]). (**D**) Top-down liquid exfoliation methods or bottom-up self-assembly approach can be used to generate SF nanofibrils (reproduced with permission [53,54]). (**E**) Advanced manufacturing techniques can be applied to fabricate complex SF-based structures such as microneedles (reproduced with permission [55]).

Silk fibroin-based materials could be generated using a variety of techniques, depending on the desired material formats [47] (Figure 3). However, regardless of the preparation method, the raw silk cocoon must first undergo degumming to eliminate sericin. Therefore, the raw silk cocoons are boiled in a diluted solution of sodium carbonate (Na_2_CO_3_) followed by rinsing and washing with pure water and overnight drying to obtain fibroin filaments. The boiling time and Na_2_CO_3_ concentration should be well controlled to prevent any negative effects this process might have on the resulting fibroin [48]. High Na_2_CO_3_ concentration and prolonged boiling time could lead to the cleavage of the disulfide bonds between H- and L-fibroin and the fragmentation of the amorphous sequences could generate fibroin of polydispersed molecular weight [49]. The most commonly utilized strategy for the preparation of silk-based materials is the dissolution of SF threads in pure fibroin solution followed by the regeneration of SF into various material formats [4]. The resulting fibroin is termed regenerated SF. In this paradigm, an appropriate solvent system, such as lithium bromide aqueous solution, Ajisawa’s reagent (calcium chloride:ethanol:water with 1:8:2 molar ratio), lithium thiocyanate aqueous solution, calcium nitrate aqueous solution, *N*-methyl morpholine-*N* oxide, hexafluoroisopropanol (HFIP), or ionic liquids, is used to dissolve SF. Each solvent system presents different solubility power, and thus they require different dissolving times and temperatures [34]. Afterward, the electrolytes are typically removed via dialysis against pure water, and aqueous solutions of fibroin are obtained. For this step, an aqueous solution of polyethylene glycol (PEG) 20 wt% could be used instead of pure water to obtain more concentrated fibroin solution. Depending on its concentration, the fibroin solution obtained after dialysis could be stored at 4 °C for months or at room temperature for weeks. This aqueous fibroin solution could be used as feedstock to generate novel SF-based materials (Figure 3A). Depending on the end-use material formats, such as film, hydrogel, particle, fiber, or scaffold, the regeneration of fibroin can be performed using different processes (Figure 3B).

Silk fibroin film can be easily generated using casting, spin-coating, or layer-by-layer methods [56,57,58]. For example, non-patterned SF film can be obtained by simply depositing a SF solution onto a plate followed by drying overnight. Subsequently, β-sheet crystalline transformation can be improved by immersing the film in an alcohol (methanol or ethanol) solution or via water annealing using a vacuum desiccator [26]. Conversely, patterned SF film can be obtained by placing a pre-formed polydimethylsiloxane (PDMS) mold into a Petri dish before fibroin deposition, drying, and β-sheet induction. Subsequently, the SF film is peeled from the PDMS mold using tweezers.

Water-containing three-dimensional (3D) networks of SF are termed SF hydrogel [59]. Physically crosslinked SF hydrogel can be produced via the transition process from liquid-like sol state to solid-like gel state. The underlying mechanism of this process is the self-assembly of proteins via enhanced hydrophobic interactions. The self-assembly gelation process can occur naturally but is usually lengthy (three months at 37 °C). To accelerate the gelation process, various stimulating tools can be used, including low pH conditions, high temperature, vortexing, ultrasonication, electrical current, lyophilization, changes in ion concentration, and dehydrating agents [60]. In addition, chemically crosslinked SF hydrogel can be generated using various chemical reactions of functionalized silk-based and other precursors, depending on the specific design. The most commonly used crosslinking approach is the enzyme-catalyzed reaction of the Tyr groups of the fibroin chain using hydrogen peroxide and horseradish peroxidase (HRP) [61].

Silk fibroin fibers are usually prepared via wet-spinning, dry jet spinning, and electrospinning processes [62]. While wet and dry spinning methods yield micro-diameter fibers, electrospinning can generate submicron- to nano-diameter fibers with extremely large surface areas and the ability to incorporate nano-sized molecules onto them. Generally, spinning dope is prepared using highly concentrated SF solutions (25%), and therefore, silk proteins can self-assemble into micelles via hydrophobic interactions and hydrogen bonding. Afterward, the resultant micelles could align and form fibers via shear stress and dehydration. Electrospun SF can be produced by applying a positive voltage to a SF solution-loaded syringe to initiate a jet aimed toward a grounded collector plate. Then, the resultant SF fibers/mats are treated with methanol followed by washing them with water to trigger the β-sheet transformation [63].

Silk fibroin can form porous 3D structures, namely sponges, foams, or scaffolds, which could be used for biomedical applications, such as tissue engineering, implantable devices, and disease models. Several techniques could be used to fabricate 3D SF sponges: salt-leaching, gas foaming, and freeze-drying [64]. The salt-leaching method can be used to obtain aqueous and HFIP-based SF sponges, which present different mechanical strengths, surface smoothness, interconnectivity, and degradation rates [47]. The general process for the fabrication of aqueous silk foam involves these key steps: pouring and evenly distributing salt into the SF solution, allowing the mixture to transform into gel, and removing the salt by immersing the mixture in water to obtain the salt-free 3D porous scaffold. Conversely, HFIP-based SF sponges can be prepared by dissolving silk in HFIP, pouring the silk/HFIP mixture over salt-containing glass, evaporating HFIP, treating the mixture with methanol, and removing the methanol and salt during the water-immersion step.

Silk fibroin microspheres/nanoparticles, which are useful for drug/protein/gene intravenous delivery systems, can be generated using the self-assembly method, lipid-based emulsifiers, or polymer co-carriers. First, SF micelles can be self-assembled via the arrangement of hydrophilic and hydrophobic chain segments of the SF molecules when ethanol is added to them, followed by quenching below their freezing point [65]. During the fabrication of lipid-based silk microspheres, a lipid (e.g., 1,2-dioleoyl-sn-glycero-3-phosphocholine) film is prepared in a tube and is used to emulsify the dropped silk/payload solution [66]. After removing water using freezing/thawing cycles and lyophilization, the lipid-coated silk vesicles are suspended in methanol and centrifuged to remove the lipid; the end products are SF microspheres. Furthermore, SF microspheres can be prepared using a more simple, water-based approach: phase separation using polymers, such as polyvinyl alcohol (PVA) [67]. The silk-PVA mixture is subjected to ultrasonication to induce phase separation. Subsequently, silk/PVA film is prepared and is dissolved before centrifuging to remove PVA and obtain SF microspheres. Various SF particulate structures can be obtained via spray drying, milling, laminar jet break-up, electrospraying, and microfluidics techniques [68,69,70,71,72].

The above-mentioned materials could only be prepared from fibroin solution after dissolving SF into molecular-scale particles. This could be unwanted when the natural mesoarchitecture and properties originate from the nanosize effect, particularly when ultra-high specific surface areas, length-to-diameter ratio, nanoporous structure, nanoconfinement effect, and optical transparency, are desired [19]. Therefore, the isolation and regeneration of SF nanofibrils could be desirable under such circumstances. Typically, SF nanofibrils could be obtained using bottom-up self-assembly approaches or top-down liquid exfoliation methods (Figure 3D). The bottom-up process consists of the dissolution of SF into fibroin solution and the stimulation of the self-assembly of SF micelles into nanofibrils by adding ethanol or simply heating the mixture [73,74]. Conversely, the top-down liquid exfoliation of silk into microfibrils/nanofibrils could be performed via ultrasonication, dissolution in calcium chloride–formic acid solution, lithium bromide–formic acid solution, HFIP solution, sodium hypochlorite aqueous solution, or combinations of these solvents [54,75,76,77,78]_._ The top-down approaches usually result in heterogeneous fibril size but higher mechanical strength, while the bottom-up methods provide homogeneous fibril size and lower mechanical strength.

Although native silk materials possess superior biocompatibility and excellent mechanical strength, they rarely meet the demands for specific applications. Owing to the advancements in physical, chemical, and genetic engineering, silks could be subjected to multi-level functionalization and modification to achieve desired properties (Figure 3C) [31]. Chemical modification via reactive amino acid groups of the silk polymer chain could be a promising solution to obtain silk-derivatives as well as conjugation products that feature photosensitivity, cell attachment ability, and anti-adhesion ability [16,79]. Genetic engineering is a strategy used to modify the structure of silk proteins and customize their properties. For example, transgenic silkworms can produce silk with remarkable fluorescence or recombinant spider silk fibers can be obtained at large-scale [80,81,82]. Feeding can be potentially used for the large-scale production of silk with enhanced properties. Using this approach, a variety of fluorescent dyes or functional nanomaterials, such as graphene, carbon nanotubes, copper nanoparticles, silver nanoparticles, and titanium dioxide can be intrinsically incorporated into silk fibers, by simply nourishing silkworms with special diets that contain these specific materials [83,84,85,86]. In addition, functional molecules can also be incorporated into silk via doping. Hence, silk with different functions, such as mechanically sensitive color-changing and near infrared (NIR) sensitivity for phototherapy can be produced via doping with stimuli-sensitive molecules, including polydiacetylene, gold nanoparticles, quantum dots, and enzymes [87,88,89,90]. Lastly, macroscopic mixing could be a simple yet powerful method for manufacturing silk-based composites with tailored characteristics. Numerous SF-based composites have been developed by combining SF with complementary biopolymers, such as collagen, chitosan (CS), gelatin, keratin, alginate, and cellulose or by mixing SF with reinforcing materials, including carbon-nanotubes, graphene oxide, and hydroxyapatite (HA) [91,92,93,94,95,96,97,98,99].

In addition to multi-level modification, to produce SF-based biomaterials with more complex architecture, advanced manufacturing tools are required. Recently, innovative state-of-the-art nanomanufacturing techniques have allowed researchers to make great progress in fabricating materials with precise structure control from nanoscale to macroscale (Figure 3E). Of all of the top-down fabrication methods, electron-beam lithography could be used to generate nanopatterns on SF film via water-solubility transition, which involves the electron-induced changing of secondary structures, followed by “water development” [100]. In addition, ion-beam lithography writing could be considered as a type of solid lithography with faster and higher resolution than electron-beam lithography for creating SF nanoscale structures from SF [101]. Additionally, nanoimprinting lithography has been used to rapidly construct nanopatterned SF film at low pressure and high resolution at moderate or elevated temperatures [102]. Soft lithography has been utilized as an effective, simple, inexpensive, and green liquid lithography method to prepare SF nanostructured film [103]. Furthermore, multiphoton lithography using femtosecond laser direct writing could be a viable option for generating arbitrary fine two-dimensional (2D)/3D nanostructures from SF in the presence of photosensitizers [104]. The self-assembly of SF assisted by poly(methyl methacrylate) or polystyrene opal templates is an example of bottom-up strategy that was used to generate inverse opal structures after etching with acetone or toluene [105,106]. Compared with complex lithography manufacturing techniques that could present possible harmful effects attributed to the harsh processing conditions, piezoelectric-based inkjet-bioprinting provides a simple, fast, and versatile method for building patterned structures from functional silk inks using a bio-friendly aqueous process [90]. Moreover, 3D printing employing extrusion dispensing devices could be exploited as an effective method for creating 3D silk-based scaffolds using functional or cell-preloaded silk bioinks that could be potentially utilized for large-scale production [107,108].

## 4. Biomedical Applications of SF-Based Materials

**Figure 4:**
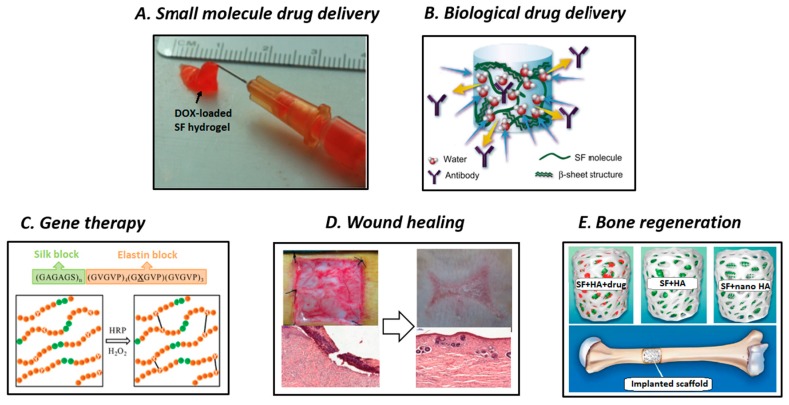
With favorable characteristics and properties, SF-based materials have been widely applied for important biomedical applications including small molecule drug delivery, biological drug delivery, gene therapy, wound healing, and bone regeneration. (**A**) For example, doxorubicin-loaded shear-thinning hydrogel can be prepared by SF aqueous solution (reproduced with permission [109]). (**B**) An entrapped monoclonal antibody can be released after water penetrating into the lyophilized SF network (reproduced with permission [110]). (**C**) Chemically crosslinked hydrogel from silk-elastin like protein is applied for gene therapy (reproduced with permission [111]). (**D**) Positive in vivo wound healing efficacy can be achieved using SF films (reproduced with permission [112]). (**E**) Scaffolds from SF and hydroxyapatite (HA) with/without drug are used for bone regeneration (reproduced with permission [113]).

Owing to their hierarchical structure and diverse range of attractive properties, SF-based materials could be good candidates for more advanced applications other than their traditional use in the textile industry, including electronics and optoelectronics, optics and photonics, water/oil filtration, and biomaterials [4,12,15,16]. Analyzing the past and current utilization of SF-based materials as biomaterials, we have noticed the routine clinical use of silk suture for wound ligation, silk fabrics for the treatment of dermatological conditions, silk surgical mesh for abdominal wall reconstruction and investigative plastic surgery applications, and also human clinical trials of SF film for wound healing or tympanic membrane perforation repairs [29]. Nonetheless, in this main section, we focused on proof-of-concept investigations and preclinical studies of SF-based novel materials in important areas of emerging biomedical applications, including small molecule drug delivery, biological drug delivery, gene therapy, wound healing, and bone regeneration (Figure 4).

Since the first Food and Drug Administration (FDA) approval in 1990, controlled DDSs have gained the interests of researchers in the academia and industrial sector [114]. Drug delivery systems can improve drug bioavailability, retain effective drug concentration, and diminish side effects compared with traditional medicine. Particularly, desirable biomaterials for DDSs should be carefully designed to: (a) allow the adequate incorporation of therapeutics, (b) protect the payloads from damage in vivo and maintain their bioactivity, (c) release the bioactive agents with predictable profile over the course of therapeutic period, and (d) biocompatible and degrade with non-toxic by-products [115]. Important FDA approved DDSs mainly include liposomes, PEGylated drug conjugates, PLGA-based systems, and protein-based systems [116]. Recently, considerable attention has been devoted to naturally occurring polymer-based DDSs owing to their excellent biocompatibility and favorable pharmacokinetics. Among various classes of biopolymers, SF-based materials are excellent choices for the delivery of bioactive molecules owing to their rare combination of robust mechanical properties, controllable biodegradability, aqueous-based purification/processing and drug stabilization effect. Typically, after incorporating them into the SF network, therapeutic agents are maintained, transported to the target sites, and released in a controlled manner [18]. In detail, the first step is loading the bioactive agents via bulk mixing, surface coating, or chemical conjugation. Then, the payloads are stabilized within the fibroin network mostly via hydrophobic interactions and/or hydrogen bonding. Subsequently, the entire system is transported to the target site using localized, systemic, or intracellular delivery routes. The β-sheet crystalline domains act as mechanical barrier; consequently the bioactive agents are diffused through the semi-amorphous regions, and the result is a sustained release profile with reduced initial burst. The release behavior can be precisely controlled by controlling the content of crystalline phase or the breaking rate of labile bonds of the conjugated SF system. The interaction between SF and drugs can be carefully manipulated, and near-zero order sustained release behavior can be achieved via diffusion- and degradation-driven mechanisms. In this section, we introduce examples of SF-based biomaterials for the delivery of therapeutic drugs and biological molecules.

Owing to their drug stabilization ability, SF-based materials have been widely investigated for the delivery of anticancer agents, which is one of the most important topics in biomedicine research. Given the hydrophobic interaction with the β-sheet crystallites in the fibroin network, poorly water-soluble anticancer drugs can be stabilized, maintain their bioactivity after being released, and improve therapy outcomes. SF-based systems have been manufactured for intratumoral delivery and also intravenous administration [117] because of their various available formats. Seib et al. used a SF-based hydrogel to deliver doxorubicin (DOX) for the localized treatment of primary breast cancer (Figure 4A) [109]. Aqueous solutions of SF were mixed with DOX before they self-assembled into a thixotropic hydrogel via sonication. After being locally injected into breast cancer-bearing rats, the loaded DOX could be controllably released from the SF hydrogel, and exhibited excellent tumor regression response and reduced metastatic spread. Wu et al. prepared DOX-loaded SF hydrogel and subjected it to a concentration-dilution process to induce the formation of nanofibers [118]. This system exhibited pH-responsive and concentration-depending release of DOX, and thus, could represent a promising tool for controlling antitumor activity. In addition to chemotherapy with anticancer drugs, phototherapy is considered to be an effective treatment method for eradicating tumors. For this approach, SF-based hydrogels were loaded with complexes of nano-graphene oxide and lanthanide-doped rare earth nanoparticles to combine their photothermal effect and upconversion luminescence imaging ability, respectively [119]. Under exposure to NIR laser radiation, the fibroin-based theranostic hydrogels greatly reduced the size of the treated tumors.

In addition to hydrogel systems for the local treatment of cancer, SF-based drug vehicles in the form of nanoparticles have been increasingly developed and investigated for their potential use for intravenous, systemic drug delivery for cancer therapy [120]. The incorporation of bioactive agents within micro-/nanoparticles could provide a tool for tuning the pharmacokinetic and biodistribution of the payload by controlling the characteristics of the carrier. Moreover, the tumor-targeting ability of the anticancer agents released from nanosized cargo would be beneficial for enhanced permeability and retention effect. Qu et al. introduced cisplatin-entrapped SF nanoparticles generated via electrospraying [121]. As a result of its strong metal-ligands coordinate bonds with the SF matrix, cisplatin could be sustainedly released for up to 15 days, and it exhibited facile intracellular penetration and enhanced inhibitory effect on a model of lung cancer cells. Furthermore, Tian et al. have prepared DOX-loaded SF-based nanoparticles with magnetic tumor targeting ability [122]. The one-pot salting out technique was used to prepare the combined SF and superparamagnetic Fe_3_O_4_ nanoparticle system. The delivery of DOX was performed using external magnetic guidance, and thus, good antitumor efficacy was achieved against multidrug-resistant MCF-7/ADR tumors with survival rate up to 100% within 30 days. In addition to the delivery of chemotherapeutic agents, such as DOX, paclitaxel, and cisplatin, researchers have become increasingly interested in the delivery of natural potential antitumor compounds, such as curcumin, using SF-based biomaterials [123,124,125]. For example, Song et al. used a strategy similar to that employed by Qu et al. [121] to prepare curcumin-encapsulated magnetic SF nanoparticles via one-pot sodium phosphate salting out [124]. Nevertheless, although the system exhibited good cellular uptake and remarkable growth inhibition toward human breast adeno-carcinoma MDA-MB-231 cells, its in vivo antitumor efficacy has not been demonstrated.

Beyond using hydrogels and nanoparticles as DDSs, tremendous efforts have been devoted to implementing other forms of SF-based biomaterials as drug delivery platforms. For instance, SF-based coatings have been studied as potential DDSs for small molecule drugs and also for biological agents. Bayraktar et al. directly deposited a SF coating formulation onto theophylline tablets [126]. The coating was formed using (1-ethyl-3-(3-dimethylamino) propyl carbodiimide-activated crosslinked SF with different PEG concentrations. Almost zero-order release profiles with prolonged theophylline release time were achieved regardless of the number of coating layers. A similar paradigm was implemented for the sustained release of adenosine [127]. The number of coating layers, SF concentration, and methanol treatment could be used as a toolkit for precisely regulating the cumulative release of adenosine. The reported SF-based coating presented remarkable potential for the treatment of pharmacoresistant epilepsy using adenosine augmentation therapy. Instead of outer coating, SF aqueous solution could also be coated simultaneously with the payloads onto substrates via stepwise layer-by-layer deposition [128]. Using the developed SF-based multilayer nanoscale coating, the controlled release of Rhodamine B, Evans blue and azoalbumin could be manipulated by adjusting the crystallinity of SF and thickness of the coating layers. For example, the sustained release of Rhodamine B could be extended from 15 to 40 days by changing the concentration of SF and using methanol treatment. Moreover, the amount of immobilized agent could be regulated by adjusting the concentration of the solution, dipping conditions, and using different methods. Cheema et al. coated SF onto an emodin-loaded liposome, which could be beneficial for tuning the release of emodin by changing the swelling and diffusion-driven release into diffusion-driven release [129]. Consequently, the system not only maintained cell specificity but also achieved improved cellular uptake; moreover the intracellular retention time of emodin was prolonged, and thus better overall drug efficacy was achieved. In addition to SF coating, Hofmann et al. reported the use of methanol-treated SF films as controlled-release drug delivery depots [130]. Increasing the degree of crystallinity of SF led to a suppressed initial burst and prolonged release of dextran of different molecular weights, which was used as model polysaccharide; similar results were obtained for HRP and lysozyme, which were used as bioactive protein models. The activity of lysozyme in methanol treated samples was better tuned than those of the control and water-treated ones. In 2008, Wenk et al. fabricated SF-based microparticulate spheres under very mild conditions using a laminar jet break-up device [70]. The diameter of the resulting microspheres was smaller than 500 μm, and the microspheres presented controllable β-sheet crystallinity degree. However, owing to the densely compacted structure obtained via crystallization with methanol or water vapor, the size, surface morphology, encapsulation efficiency, and drug release behavior of the treated SF microspheres changed. Using these SF microspheres, different in vitro release rates and fashions were obtained for salicylic acid, propranolol HCl and insulin-like growth factor I (IGF-I), which could be attributed to the different methanol solubility, hydrophobicity, pKa values, and molecular weights of the payloads. In their comprehensive study Pritchard et al. analyzed the utility of various SF-based biomaterial formats, including bulk-loaded silk films, 3D porous sponges, nanofilm coatings, bulk hydrogels, microsphere-loaded hydrogels, and degummed silk fibers, for focal antibiotics delivery [131]. Several antibiotics, including penicillin, ampicillin, gentamicin, cefazolin, rifampicin, and erythromycin were effectively loaded and delivered at tunable release rates using various SF-based biomaterial formats. Furthermore, the growth of *Staphylococcus aureus* (*S. aureus*) lawn was repressed using rifampicin-loaded SFs and the in vivo wound healing ability of ampicillin-loaded SF hydrogels on BALB/c mice models was confirmed to be effective; this demonstrated the promising feasibility of antibiotic-releasing SF-based materials for preventing infections and aiding wound healing. Utilizing the sophisticated micro-molding technique, an SF-based microneedle device was manufactured and used as a safe and pain-free transdermal delivery system [55]. Owing to its unique combination of favorable mechanical strength and ability to maintain the activity of encapsulated bioactive factors, the SF-based microneedle device was demonstrated to be an ideal alternative to other material-based microneedles for the delivery of sensitive biological molecules. The sufficient penetration into mouse skin, the controlled release of HRP while maintaining its activity, and also the infection reduction ability of the tetracycline-loaded SF-based microneedles confirmed the feasibility of SF-based materials for minimally invasive DDSs.

In addition to the delivery of small molecule drugs, the delivery of biological molecules, such as proteins, growth factors, peptides, and genes is also an important area of biomedical applications. Environmentally sensitive molecules are usually unstable and tend to degrade or aggregate during processing. Therefore, SF could be an excellent candidate for immobilizing and preserving biological molecules owing to its properties, particularly its tunable insolubility and very mild processing. The advantageous stabilization effect and mechanism of SF biomaterials toward biologics, including enzymes, antibiotics, vaccines, and plasma molecules have been discussed in detail by Li et al. [27]. Mandal et al. developed a semi-interpenetrating network hydrogel of fibroin/polyacrylamide (PAAm), as an example of SF-based protein delivery system for the delivery of fluorescein isothiocyanate (FITC)-labelled insulin [132]. The swelling and mechanical properties of this interpenetrating polymer network hydrogel were increased by incorporating and crosslinking PAAm simultaneously with fibroin. The SF/PAAm ratio could be used to manipulate the degradation, swelling properties, and payload release behavior. In addition to its utilization as the main matrix of composite hydrogels, SF can also be used as reinforcing electrospun mesh for the preparation of silk-hyaluronic acid composite hydrogels [133]. The vascular endothelial growth factor (VEGF) was used as cytokine model and was non-covalently encapsulated into composite gels prior to crosslinking it in the presence of polyethylene glycol diacrylate. The addition of 0.3% thiol-modified heparin to the release medium led to the increase in the percent release of VEGF from 25% to 57% over 42 days. The developed mat-reinforced composite structure allowed to simply secure the implanted hydrogel disk in the surrounding tissue or organs using sutures or staples. Guziewicz et al. reported that therapeutic monoclonal antibodies could be maintained and released from a SF lyogel over extended periods [110]. The high density of the β-sheet network of the lyophilized gels was the main driving force that allowed the prolonged release of the loaded antibodies (Figure 4B). Furthermore, the report presented a simple yet convenient approach for entrapping and storing therapeutic proteins within the SF matrix while achieving maximum long-term stability of proteins. Uebersax et al. fabricated a biodegradable, nerve growth factor (NGF)-embedded nerve conduit from SF solution using different preparation methods, including shock-freeze drying, slow lyophilization, and air drying [134]. Afterward, it was demonstrated that the proliferation and differentiation of PC12 cells and improvement in the outgrowth of neurite upon stimulation with NGF conferred by SF-based materials was comparable with that provided by collagen or laminin coatings. Moreover, owing to the strong ionic interaction with the SF matrix, the release of NGF could be controlled over more than three weeks and its very low total released percentage ranged from 0.3% to 13.0%. In 2010, Wenk et al. presented an approach for decorating growth factor binding sulfonate moieties onto SF molecules via diazonium coupling addition [135]. The resulting functionalized SF-derivative film exhibited an increased binding affinity for fibroblast growth factor 2 (FGF-2) owing to the strong ionic interactions between them. The binding with FGF-2 varied almost linearly with the degree of sulfonation, which is appealing for controlling the delivery of FGF-2 under various tissue engineering conditions. Lastly, the decrease in the metabolic activity of human mesenchymal cells and the increase in the levels of phosphorylated extracellular signal-regulated kinases (pERK1/2) was observed, which indicated that the loaded growth factor retained its potency. Lovett et al. studied the controlled delivery of the anti-vascular endothelial growth factor (anti VEGF), namely bevacizumab or Avastin that was aimed at treating age-related macular degeneration, using a SF hydrogel system [136]. In vitro release kinetics measurements suggested that the sustained release of the loaded angiogenesis inhibitor could be achieved for at least three months. Consequently, the standard dose- and high dose-loaded hydrogel formulations provided therapeutic levels of bevacizumab in both the vitreous and aqueous humors for at least three times longer than the control group of solution after rabbit models were injected with bevacizumab intravitreally. The mean half-life of bevacizumab in the vitreous and aqueous humors was much higher when hydrogel formulations were used compared with that observed for the standard dose control group. These results confirmed the applicability of SF for the long-term delivery of bevacizumab and other anti-VEGF therapeutics. Wang et al. reported the use of SF for the fabrication of a protein delivery system [51]. In their study, the SF outer shell was deposited on PLGA or alginate microspheres via layer-by-layer coating using different penetration methods. The SF outer coating not only improved the degradation stability of the resulting microspheres but also significantly decreased the initial burst and prolonged the release of encapsulated HRP and Rhodamine-labeled bovine serum albumin from PLGA and alginate, respectively. In scaffold form, SF could also serve as a depot for the controlled release of proteins, as reported by Uebersax et al. [137]. In their study, IGF-I was simply incorporated with SF solution before generating the scaffold and was released in a sustained manner over 25 days using pH, methanol treatment, and payload concentration-depending kinetics. After culturing human bone marrow-derived mesenchymal stem cells (hMSCs) with IGF-I-loaded SF scaffold in insulin-free transforming growth factor beta 1 supplemented medium, the in vitro chondrogenic differentiation of hMSCs was observed within three weeks, which demonstrated their potential feasibility for cartilage regeneration. Recently, microneedle vaccination has been of special interest in biomedicine research owing to the painless, non-invasive delivery of vaccines to enhance immunity. Silk-poly(acrylic acid) (PAA) composite microneedles were developed and used to control vaccine delivery kinetics and achieve good immunity efficacy in vivo [138]. Using the tip-pedestal composite design, where ovalbumin-loaded SF hydrogel tips were combined with ovalbumin-loaded PAA pedestals, the silk solid tip could be implanted rapidly and retained persistently after the removal of the PAA pedestal and prolonged the release of the loaded adjuvant. The in vivo use of vaccine-loaded microneedles on the auricular skin of mice led to the rapid clearance of the ovalbumin released from PAA or intradermal injection within 24 h while the prolonged release of ovalbumin for 5 and 16 days was observed for the untreated and methanol-treated silk-implant groups, respectively. The in vivo findings together with the long-term storage ability suggested the promising potency of silk-PAA microneedles for programmable vaccine delivery that is intended to improve overall immunity.

In contrast with the previously mentioned SF-based systems where the biological and physiochemical properties are generally tailored using the processing conditions, crystallinity, protein molecular weight, chemical modification, and physical mixing with different materials, genetically engineered SF with precise composition, sequence, and block length could provide high level of control over functions and performance and hence, could represent tailored options for particular biomedical needs [139]. Moreover, the incorporation of functional motifs that regulate self-assembly, stimuli-sensitivity, biorecognition, and biodegradability can be achieved using such materials. Recent gene therapy research studies have extensively used bioengineered silk-elastin-like polymers (SELPs) as carriers for the delivery of plasmid DNA or adenoviral vectors (Figure 4C). The chemical structure of SELPS comprises tandemly repeated units of silk-like (Gly–Ala–Gly–Ala–Gly–Ser) and elastin-like (Gly–Val–Gly–Val–Pro) blocks. Silk-elastin-like polymers were proposed because they combine the heat and mechanical stability of semi-crystalline SF blocks with the water solubility and flexibility of elastomeric elastin blocks. Moreover, the manipulation of the monomer structure at genetic level and adenoviral viability are special benefits of this material that render it suitable for gene therapy. Li et al. reported the delivery of plasmid DNAs of varied molecular weights and models of adenoviral vectors using SELP hydrogels [140]. Encapsulated plasmid DNAs exhibited release kinetics that were governed by the molecular weight and conformation of the DNA and geometry of the hydrogel and presented no significant loss of bioactivity over 28 days. The in vivo intratumoral delivery of *Renilla* luciferase plasmid using SELP hydrogel in a breast cancer-bearing mouse model exhibited a significant increase in the gene expression of luciferase and retention of transfection efficacy, which was preferable within the tumor. Hatefi et al. further analyzed the in vitro and in vivo adenoviral delivery using SELPs [141]. The in vitro sustained release of entrapped adenoviruses with maintained infection activity was observed within 28 days and was attributed to the stabilization effect between the silk or elastin units and virus. As a consequence of in vivo tumoral injections in xenograft murine models, green fluorescent expression (GFP) was prolonged even after 15 days using virus-loaded SELP samples, while a decrease in GFP expression could be observed after 11 days for the virus injection alone; this rendered these polymers useful for local adenovirus controlled delivery for cancer treatment. Subsequently, Ghandehari et al. examined the anticancer efficacy of an adenovirus-incorporated SELP hydrogel in a murine xenograft model of head and neck cancer [142]. After 14 days of monitoring, the antitumor effect of the adenovirus-incorporated SELP hydrogel was significantly higher than that of the adenovirus/ganciclovir alone. The results of a large number of recent investigations on the utility, modification, and fabrication of different SELPs have been positive. Huang et al. thoroughly reviewed SELP-based biomaterials for the development of various stimuli-sensitive systems, analyzed their sequence-structure-function relationship, and examined their current or potential applications [139].

Wound healing is a complex and dynamic process that involves a series of consecutive steps: hemostasis, inflammation, proliferation, and remodeling. Current commercially available wound dressing materials present several drawbacks, including poor mechanical strength, high cost, invariability of collagens, low processability, limited availability of elastins, and non-biodegradability of soft silicons and polyurethanes. Therefore, SF could be considered to be an exceptional wound healing material owing to its abundance, intrinsic biodegradability, biocompatibility, mechanical robustness, signaling molecules stabilization ability, high water and oxygen uptake, and low immunogenicity. In addition to the above-mentioned properties, SF also promotes healing via more complicated action mechanisms and signaling pathways, which have been briefly summarized by Farokhi et al. [143]. Of various material formats, researchers focused their attention on electrospun SF nanofiber scaffold owing to its favorable nanoscale fiber structure which is able to retain water and allows sufficient oxygen permeation or fluid drainage while still protecting the scaffold from outer microorganisms. Uttayarat et al. coated an SF electrospun nonwoven mat with gamma radiation-prepared colloidal silver nanoparticles (AgNP) to confer microbacterial properties to the resulting material [144]. This material presented similar anti-bacterial ability against *S. Aureus* and *Pseudomonas aeruginosa* (*P. Aeruginosa*); moreover the inhibition effect of AgNP-SF in comparison with commercial wound dressing while possessing an approximately 20 times lower silver concentration. In addition to incorporating antimicrobial agents to prevent the growth of harmful bacteria, wound dressing matrices are usually loaded with growth factors to promote epithelization. Schneider et al. evaluated the wound healing abilities of epidermal growth factor (EGF)-loaded electrospun silk mats on human skin-equivalent models [145]. After 48 h, the wound closure percentage of the EGF-silk group reached 98.5%, compared with only 8.9% and 26.3% for the control and silk only groups, respectively. The burst release of EGF from the surface of silk nanofibers was an essential requirement for the first stage of wound closure and contributed to the great reepithelialization results on the human skin model. In a related study aimed to evaluate the feasibility of EGF/silver sulfadiazine/SF systems on wound dressing applications, Gil et al. investigated the effect of different SF material formats and drug loading methods on wound healing in vivo [146]. Silk-based films, lamellar porous films, and electrospun mats were loaded or coated with EGF/silver sulfadiazine and used as wound dressing on BALB/C mice. On visual examination, the wound healing effects of these systems were comparable and sometimes healing occurred faster and with less scar formation than that induced in the positive control Hydrocolloid^®^ dressing group. Moreover, histological tests also reinforced the in vivo findings by demonstrating that SF decreased the number of inflammatory cells and provided structural support for cell adherence, proliferation, and migration. Recently, antimicrobial peptides have received great interest as a promising solution for overcoming the low efficacy or adverse effects of common antimicrobial agents. Recently, Woong et al. immobilized an antimicrobial peptide, Cys-KR12, onto a SF nanofiber membrane for wound healing applications [147]. The peptide was chemically immobilized onto SF using the thiol-maleimide Michael addition reaction, which could decrease its cytotoxicity and increase its stability. The incorporation of the Cys-KR12 peptide onto the SF membrane helped to prolong the suppression of *S. Aureus*, *Staphylococcus epidermidis*, *Escherichia coli*, and *P. aeruginosa* growth, and improve metabolic activity and DNA content of cultured HaCaT and normal human dermal fibroblast cells, and support the proliferation and differentiation of keratinocytes, which is important for the final stage of wound healing. In addition to SF electrospun mats with rather simple structure, multicomponent SF-based composite systems have been widely researched. Insulin-loaded SF microspheres encapsulated within SF sponges have been reported for the treatment of chronic cutaneous wounds [148]. Owing to the stimulatory effect of insulin, keratinocytes and endothelial cells could exhibit increased proliferation and differentiation. Bioactive insulin was encapsulated within SF microspheres via coaxial electrospraying and lyophilization, followed by the multilayer loading of microparticles into the SF solution prior to freeze-drying to form sponge composite structures. The released insulin was able to maintain its bioactivity and also accelerate in vivo wound healing in streptozotocin-induced diabetic rats by promoting vascularization via stimulating endothelial cell viability. Kim et al. reported the use of SF-calcium alginate-carboxymethyl cellulose hydrogel, which is an SF-based blending composite, for the treatment of burn wounds [149]. After 21 days of treatment, the SF composite hydrogel provided better cytotoxicity and comparable wound healing effect to Purilon Gel^®^, which was used as positive control. Moreover, Vasconcelos et al. developed a blending hydrogel system that combined the flexibility, elasticity, and swelling ability of elastin with the tunable biodegradation and high mechanical strength of silk and used it as wound dressing material [150]. Genipin crosslinked elastin/silk healed burn wounds faster than commercial collagen dressing, as it only required six days of treatment. Chitosan, a naturally occurring, abundant polysaccharide with excellent antibacterial properties, has been incorporated with SF in recent studies that have targeted its potential for wound dressing applications. Cai et al. obtained an electrospun nanofiber composite from blending a mixture of SF and CS solutions in HFIP and HFIP/trifluoroacetic acid solvent, respectively [151]. In another study that aimed to eliminate toxic organic solvents or acids from electrospun materials, CS was functionalized with acrylic acid groups to yield water-soluble N-carboxyethyl chitosan, followed by blending with SF and PVA in an aqueous solution [152]. To improve stability and durability of the CS/SF blend, Gu et al. performed chemical crosslinking using alginate dialdehyde [153]. Although many attempts were made to develop SF-based biomaterials for wound dressing, the lack of safety and efficacy data in large animal models or human clinical trials has been a big challenge to date. Zhang et al. conducted a rare, translational research, where clinically oriented, comprehensive preclinical studies were performed on rabbit and porcine full-thickness skin defect models and randomized controlled clinical trials were carried out on human subjects [112]. The SF film was fabricated following a simple procedure, by air drying an aqueous solution of SF. The generated SF presented high fluid handling ability and gaseous permeability, and also waterproof surface induced via water-vapor treatment. The comprehensive and standardized in vitro biocompatibility of the SF film was evaluated in terms of cytotoxicity, intracutaneous reactivity, sensitization, acute systemic toxicity, subchronic systemic toxicity, genotoxicity, and hemolysis. Testing results demonstrated the overall biocompatibility of SF film which allowed the use of this film in clinical trials. Preclinical trials on small animal models (rabbits) resulted in fast reepithelization and remarkable and quick skin regeneration when a SF film was used in the absence of antimicrobial agents or growth factors (Figure 4D). Afterward, the long-term assessment on relevant large animal models (porcine) demonstrated the healthy and effective wound healing effect of the SF film. Lastly, in clinical trials on 71 human subjects, it was demonstrated that the healing time achieved using the SF film (9.86 days) was significantly shorter compared with that reached using Sidaiyi (11.35 days), a commercial SF sponge-silicon two layered scaffold for wound dressing with low risk of infection or exudation.

An important biomedical application of SF that we discuss in detail in this final section is bone tissue regeneration. Construction of bone tissue requires the harmonized combination of cells, supporting scaffold, and additional bioactive agents, such as growth factors or mechanical stimuli [154]. In addition to minor constituents, natural bone tissue comprises collagen as the main component of the organic extracellular matrix (ECM), HA as the primary inorganic mineral, and water [155]. The mechanical strength and hierarchical architecture of bone relies on the nanostructured arrangement of HA crystallites embedded within the collagen fibrillar matrix. During the bone formation process, the ECM mostly contributes to regulating the differentiation of encapsulated stem cells toward osteogenic lineage. Given the demand for bone tissue regeneration materials, potential material systems are typically manufactured from bioceramics, synthetic/natural polymers, or their combinations [156]. Hence, among a variety of polymers, SF has been considered to be a promising ECM-mimicking scaffold material with excellent mechanical strength, remarkable biocompatibility, non-immunogenicity, tunable degradation, and diverse modification [157]. Given its potential for a broad range of bone tissue engineering applications, an increasing number of investigations on SF, its functionalized variants, hybrid systems, and blending systems for bone scaffolds have been reported recently. Farokhi et al. investigated the SF/HA composite material, which is an important silk-based platform for bone tissue engineering [113]. In SF/HA systems, HA, which is a brittle osteogenic bioceramic, is combined with silk, which features extraordinary elasticity and flexibility, to generate ceramic/polymer hybrid systems that are very similar to natural bone in terms of structure, and thus could be used as bone constituents (Figure 4E) [158,159]. The combination of HA with silk could improve the crystal formation of HA and provide coordinative effect between the structure and properties of silk and those of HA. Silk/HA systems could induce osteogenesis and angiogenesis by stimulating the proliferation, adhesion, and differentiation of osteoblasts, and thus, could result in effective bone regeneration. These systems could be prepared as direct-written 3D scaffolds, injectable hydrogels, films, or nanoparticle-reinforced scaffolds. Sun et al. fabricated a 3D SF/HA scaffold with gradient pore sizes using the direct-writing assembly technique [160]. To construct such a scaffold, a concentrated suspension of SF/HA with appropriate viscosity was extruded through a micronozzle onto a micropositioning stage prior to methanol treatment. Co-culturing hMSCs and human mammary microvascular endothelial cells resulted in the formation of a network-like structure, which could not be observed when hMSCs were cultured alone. A cross-shape structure with endothelial characteristics was observed only at the pore size of 400 µm, which demonstrated the feasibility of this system for studying the effect of the scaffold structure toward bone tissue formation. In contrast to the previously described 3D scaffold system that could be used for implantable tissue, an injectable hydrogel system based on silk nanofibers could be useful for repairing irregular bone defects using a minimally invasive method [161]. The mechanical properties of the composite hydrogel obtained by blending water-dispersible HA (high content of up to 60 wt%) with SF nanofiber (SF-HA60) hydrogel mimicked those of the bone-like ECM while still maintaining favorable shear-thinning properties for injectability. Subsequently, better osteogenic differentiation of rat bone marrow-derived mesenchymal stem cells from the composite hydrogel was confirmed by the significantly higher degree of alkaline phosphatase activity and expression of Runx2, osteocalcin, and osteopontin observed when the composite hydrogel was used, compared with those noted when the SF hydrogel was used. Lastly, an in vivo bone reconstruction evaluation on cranial bone defect rat models was conducted over 16 weeks, and the fastest bone regeneration and highest ratio of new bone formation was observed for the SF-HA60 hydrogel. Compared with other SF/HA composite systems that used HA powder, the utilization of HA in nanocrystallite form, which featured a very high surface area, could improve densification, and could result in the remarkable fracture toughness and excellent mechanical properties of the obtained material. Mi et al. recently fabricated for the first time a series of SF nanofibril/nanoHA-based materials with homogeneous structure via the in situ mineralization of nanoHA in SF solution, followed by vacuum filtration to create hard films or centrifugation in the presence of NaCl to obtain soft hydrogels [162]. Given the thixotropic properties and adjustable mechanical strength of the hydrogels, porosity and integrity of the films, and their simple, eco-friendly preparation methods, the presented systems hold remarkable potential for bone regeneration applications. Owing to its high mechanical strength and RGD integrin-binding motif, non-mulberry tropical tasar silkworm (*Antheraea mylitta*) fibroin has been considered to be a material able to achieve superior osteoblast adherence and improved bone regeneration process compared with the widely used *B. mori* SF [163]. Various composite scaffold architectures were fabricated including HA-coated, nanoHA-reinforced, and SF-HA-nanocomposite-reinforced scaffolds. Compared with the control group (nanoHA-reinforced scaffold), the HA-coated scaffold presented superior mechanical strength, better adhesion of osteoblast, and long-term mineralization, while the SF-HA-nanocomposite-reinforced scaffold provided better conditions for the co-culturing of osteoblasts and macrophages. Despite numerous investigations on SF/HA biomaterials for bone tissue engineering, systems with advantageous self-healing properties have been seldom reported. Recently, Shi et al. described the dual crosslinked, self-healing hydrogel prepared from calcium phosphate-modified SF microfibers (Cap-mSF) and hyaluronic-based binder [164]. In this system, the dynamic metal-biphosphonate (BP) coordination between CaP-mSF and the BP-polymeric binder presented self-healing properties. Additionally, this system exhibited UV-induced photocrosslinking owing to the presence of unsaturated double bonds in the polymeric binder chain. The hydrogel-implanted group exhibited much better bone regeneration response in the rat cranial critical defect model after 8 weeks compared with the untreated group. In addition to SF/HA-based systems, SF can also be used solely or in combination with other materials for bone tissue engineering. Uebersax et al. obtained macroporous SF scaffolds from different silk sources, using two different protocols: HFIP-evaporation with NaCl porogen and ultrapure water (UPW) freeze-drying with paraffin porogen [165]. After implanting the scaffold into sheep drill hole models, new bone formation could be observed for each examined group in all areas (periphery, middle part, center) of the implanted sites; nevertheless, no significant osteoconductivity was achieved. Both SF scaffolds supported tissue infiltration and vascularization, however, UPW-SF exhibited a higher level of interconnectivity and larger diameter of interconnective pores, which could be beneficial for the fusion of bone islands toward local, trabecular-like bone clusters and for tissue ingrowth. Recombinant human BMP-2, a potent osteoinductive cytokine model, was incorporated into SF scaffolds in the presence or absence of hMSCs to investigate its possible osteogenic response for bone healing [166]. The bone formation effect of the BMP-2/SF scaffold was even greater in terms of bone volume and mechanical strength than that of the undifferentiated hMSCs supplied BMP-2/SF scaffold. The in vivo findings indicated the supportive delivery of BMP-2 by the SF scaffold healed moderate defects and the apparent potential of BMP-2/SF for the treatment of acute injury without the need for supplementary hMSCs.

## 5. Conclusions and Future Outlook

In this brief review, we have discussed and offered insights on SF, an ancient yet attractive material, for emerging biomedical applications. The ubiquity, unique hierarchical structure, robust mechanical strength, excellent biocompatibility, tunable biodegradation, versatility in material format design, and mild aqueous processing are some of the key advantageous characteristics of SF. Undoubtedly, SF plays a dominant role in biomedical applications, compared with other synthetic or natural polymers. Current advancements in nanofabrication technologies and also multilevel modifications are promising for SF-based materials with more sophisticated designs. However, to transition from academic research achievements to industrial settings, a lot of attempts should be made to developing manufacturing processes that are eco-friendly, can be easily scaled-up, present small batch-to-batch variations, are simple, and time-saving. In addition, the long-term storing ability of highly concentrated regenerated SF aqueous solution feedstock should be an important aspect. Another intrinsic limitation of natural polymers, which is the difficulty to control their structure and molecular weight, should be considered. Lastly, a large number of pre-clinical trials on appropriate animal models and clinical examinations should be performed to expand the use of SF from basic biomedical research into clinical practice. Hopefully, this ancient, luxury textile material will bring extra value to future applications and will not be limited to sericulture but will expand into modern high-tech industries. Currently, the desirable potential of SF for biomedical industries is getting elicited and becoming reality by the appearance of companies with commercialized products and ongoing clinical trials [29]. Given the harmonious collaboration between innovators from the academic and industrial sectors, we believe that the new Silk Road could connect the old textile industry with future healthcare applications with unprecedented, seemingly endless opportunities.
